# No consistent effect of cerebellar transcranial direct current stimulation on visuomotor adaptation

**DOI:** 10.1152/jn.00896.2016

**Published:** 2017-03-15

**Authors:** Roya Jalali, R. Chris Miall, Joseph M. Galea

**Affiliations:** ^1^Physical Sciences of Imaging in the Biomedical Sciences, Doctoral Training Centre, University of Birmingham, Birmingham, United Kingdom; ^2^School of Psychology, University of Birmingham, Birmingham, United Kingdom

**Keywords:** adaptation, brain stimulation, cerebellum, motor learning, tDCS

## Abstract

Cerebellar transcranial direct current stimulation (ctDCS) is known to enhance adaptation to a novel visual rotation (visuomotor adaptation), and it is suggested to hold promise as a therapeutic intervention. However, it is unknown whether this effect is robust across varying task parameters. This question is crucial if ctDCS is to be used clinically, because it must have a consistent and robust effect across a relatively wide range of behaviors. The aim of this study was to examine the effect of ctDCS on visuomotor adaptation across a wide range of task parameters that were systematically varied. Therefore, 192 young healthy individuals participated in 1 of 7 visuomotor adaptation experiments in either an anodal or sham ctDCS group. Each experiment examined whether ctDCS had a positive effect on adaptation when a unique feature of the task was altered: position of the monitor, offline tDCS, use of a tool, and perturbation schedule. Although we initially replicated the previously reported positive effect of ctDCS on visuomotor adaptation, this was not maintained during a second replication study or across a large range of varying task parameters. At the very least, this may call into question the validity of using ctDCS within a clinical context where a robust and consistent effect across behavior would be required.

**NEW & NOTEWORTHY** Cerebellar transcranial direct current stimulation (ctDCS) is known to enhance motor adaptation and thus holds promise as a therapeutic intervention. However, understanding the reliability of ctDCS across varying task parameters is crucial. To examine this, we investigated whether ctDCS enhanced visuomotor adaptation across a range of varying task parameters. We found ctDCS to have no consistent effect on visuomotor adaptation, questioning the validity of using ctDCS within a clinical context.

## Abstract (precis)

Cerebellar transcranial direct current stimulation (ctDCS) is known to enhance motor adaptation and thus holds promise as a therapeutic intervention. However, understanding the reliability of ctDCS across varying task parameters is crucial. To examine this, we investigated whether ctDCS enhanced visuomotor adaptation across a range of varying task parameters. We found ctDCS to have no consistent effect on visuomotor adaptation, questioning the validity of using ctDCS within a clinical context.

motor adaptation is a specific form of motor learning, which refers to the error reduction that occurs in response to a novel perturbation (Krakauer 2009; Shadmehr and Mussa-Ivaldi 1994). Specifically, when we make a movement with a defined goal, i.e., reaching to a visual target, the brain compares the actual and predicted sensory outcome of the executed movement. A sensory prediction error can be induced by a systematic perturbation such as a visual rotation or force field. This perturbation induces prediction errors that inform the brain of an environmental change (Miall and Wolpert 1996; Wolpert et al. 1998). To return to accurate performance, the brain gradually updates its prediction, and resulting motor commands, so that it accounts for the new dynamics of the environment (Tseng et al. 2007; Yamamoto et al. 2006).

Patients with cerebellar lesions show a pronounced impairment in their ability to adapt to novel perturbations (Criscimagna-Hemminger et al. 2010; Diedrichsen et al. 2005 Donchin et al. 2012; Martin et al. 1996; Maschke et al. 2004; Rabe et al. 2009; Smith and Shadmehr 2005; Weiner et al. 1983; Yamamoto et al. 2006). Specifically, they are often unable to reduce the movement error induced by the visual rotation or force field. This suggests that the cerebellum is crucial during the feedforward process required for successful motor adaptation. Although patient studies can provide us with a good insight regarding cerebellar function, there is a scarcity of patients with isolated cerebellar lesions. In addition, testing patients leaves the possibility that some changes, or the lack of them, are due to long-term compensation by other brain areas.

An alternative approach to investigate cerebellar function is to use noninvasive brain stimulation such as transcranial direct current stimulation (tDCS) in healthy participants. For instance, Galea et al. (2011) applied tDCS over the cerebellum (ctDCS) during adaptation to a visual rotation (visuomotor adaptation). It was found that anodal ctDCS led to faster adaptation, relative to either primary motor cortex (M1) anodal tDCS or sham tDCS (Galea et al. 2011). Such positive effects of ctDCS on cerebellar function have been replicated in visuomotor adaptation (Block and Celnik 2013; Cantarero et al. 2015; Galea et al. 2011; Hardwick and Celnik 2014), force field adaptation (Herzfeld et al. 2014), locomotor adaptation (Jayaram et al. 2012), saccade adaptation (Avila et al. 2015; Panouillères et al. 2015), motor skill learning (Cantarero et al. 2015), and language prediction tasks (Miall et al. 2016). As a result, it has been suggested that cerebellar tDCS is not only a useful tool to understand cerebellar function but also a possible clinical technique to restore cerebellar function in patients suffering cerebellum-based disorders (Grimaldi et al. 2014). However, there are also inconsistencies regarding the impact of ctDCS, with several studies reporting ctDCS having no effect on motor learning (Mamlins 2016; Steiner et al. 2016).

For ctDCS to be applied in a clinical context, we must first understand how consistent the effects of ctDCS are within a particular learning context. Therefore, we examined the influence of anodal ctDCS on visuomotor adaptation across a range of different task parameters. Specifically, we examined whether ctDCS produced a reliable behavioral effect when task parameters such as screen orientation, tDCS timing, tool use, and perturbation schedule were manipulated.

## MATERIALS AND METHODS

### 

#### Participants.

A total of 192 healthy young individuals participated in this study (120 women; 25 ± 7 yr). Each participated in one of seven experiments and received either anodal or sham ctDCS. All were blinded to the stimulation, naive to the task, self-assessed as right-handed, had normal/corrected vision, and reported to have no history of any neurological condition. The study was approved by the Ethical Review Committee of the University of Birmingham and was in accordance with the Declaration of Helsinki. Written informed consent was obtained from all participants. Participants were recruited through online advertising and received monetary compensation on completion of the study. At the end of the session, participants were asked to report their attention, fatigue, and quality of sleep using a questionnaire with a scale from 1 to 7, and also reported their perceived tDCS as active (1) or placebo (0), and their hours of sleep in the previous night (Table 1). These self-reports were collected from 164 participants, with 1 excluded from *experiments 1* and *2*, 13 (either anodal or sham) from *experiment 5*, and all 13 sham participants from *experiment 7*.

**Table 1.** Self-reported rate of attention, fatigue, and sleep

	Attention	Fatigue	Sleeping Hours	Quality of Sleep	Active or Placebo
*Experiment 1*					
Anodal	5.3 ± 1.2	4.1 ± 1.4	7.3 ± 1.6	4.6 ± 1.8	0.9 ± 0.3
Sham	4.6 ± 1.1	3.7 ± 1.5	7.2 ± 1.6	4.7 ± 1.7	0.7 ± 0.5
*t*-test	*t*_(25)_ = 1.5, *P* = 0.1	*t*_(25)_ = 0.8, *P* = 0.5	*t*_(25)_ = 0.2, *P* = 0.8	*t*_(25)_ = 0.1, *P* = 0.9	*t*_(25)_ = 1.4, *P* = 0.2
*Experiment 2*					
Anodal	5.9 ± 1	3.3 ± 1.6	7.7 ± 1.6	5.3 ± 1.1	0.9 ± 0.3
Sham	5.2 ± 1.2	3.8 ± 1.7	7.4 ± 2.8	5.4 ± 0.5	1 ± 0
*t*-test	*t*_(18)_ = 1.3, *P* = 0.2	*t*_(18)_ = 0.6, *P* = 0.5	*t*_(18)_ = 0.4, *P* = 0.7	*t*_(18)_ = 0.4, *P* = 0.7	*t*_(18)_ = 0.9, *P* = 0.4
*Experiment 3*					
Anodal	5.0 ± 1.1	3.9 ± 1.6	8.0 ± 1.0	5.3 ± 1.0	0.8 ± 0.4
Sham	5.4 ± 1.3	4.0 ± 1.5	7.4 ± 1.4	5.3 ± 1.1	0.7 ± 0.5
*t*-test	*t*_(22)_ = 0.6, *P* = 0.5	*t*_(22)_ = 0.6, *P* = 0.8	*t*_(22)_ = 0.4, *P* = 0.1	*t*_(22)_ = 0.4, *P* = 0.8	*t*_(22)_ = 0, *P* = 1.0
*Experiment 4*					
Anodal	5.6 ± 1	2.7 ± 1	6.9 ± 1.2	5.1 ± 1.2	0.9 ± 0.3
Sham	5.8 ± 1	2.8 ± 1	7.0 ± 1.3	5.0 ± 1.8	0.8 ± 0.4
*t*-test	*t*_(19)_ = 0.5, *P* = 0.6	*t*_(19)_ = 0.04, *P* = 0.9	*t*_(19)_ = 0.2, *P* = 0.8	*t*_(19)_ = 0.1, *P* = 0.9	*t*_(19)_ = 0.9, *P* = 0.4
*Experiment 5*					
Anodal	5.0 ± 0.9	3.0 ± 1.4	7.6 ± 1.0	5.3 ± 1.0	0.7 ± 0.5
Sham	5.32 ± 1.3	3.4 ± 1.5	7.3 ± 1.4	5.3 ± 1.1	0.4 ± 0.5
*t*-test	*t*_(21)_ = 0.4, *P* = 0.7	*t*_(21)_ = 0.6, *P* = 0.5	*t*_(21)_ = 0.6, *P* = 0.6	*t*_(21)_ = 0.8, *P* = 0.4	*t*_(21)_ = 1.4, *P* = 0.2
*Experiment 6*					
Anodal	5.0 ± 1.2	4.2 ± 1.6	7.8 ± 1.0	5.1 ± 1.0	0.7 ± 0.5
Sham	5.4 ± 1.0	3.5 ± 1.6	7.1 ± 1.3	5.1 ± 1.4	0.6 ± 0.5
*t*-test	*t*_(30)_ = 0.8, *P* = 0.4	*t*_(30)_ = 1.2, *P* = 0.2	*t*_(30)_ = 1.6, *P* = 0.1	*t*_(30)_ = 0, *P* = 1.0	*t*_(30)_ = 0.7, *P* = 0.5

Data are self-reported rates of attention, fatigue, sleep hours, quality of sleep (1 is poorest and 7 is the maximal), and perception of tDCS as active (1) or placebo (0). All values were averaged and compared using independent *t*-test across the whole experiments and are presented as means ± SD.

#### Experimental procedure.

**Fig. 1.**
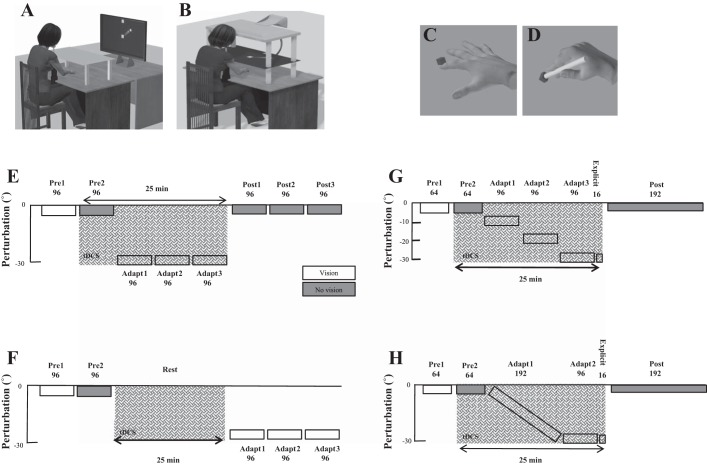
*A*: vertical screen setup. Participants sat behind a table facing a vertically orientated screen placed 105 cm in front of them. *B*: horizontal screen setup. Participants sat in front of a horizontally suspended mirror. The mirror prevented direct vision of the hand and arm but showed a reflection of a computer monitor mounted above that appeared to be in the same plane as the hand. *C*: sensor attached to finger. The initial experiment started with the Polhemus sensor attached to the right index finger. *D*: sensor attached to a pen-shaped tool. Participants were asked to hold the top part of the pen. *E*: abrupt 30° visual rotation (VR) protocol. Following 2 baseline blocks (96 trials: *pre 1* and *pre 2*), an abrupt 30° VR was applied to the screen cursor and was maintained across 3 blocks (*adapt 1–3*). ctDCS (anodal/sham) was applied from *pre 2* until *adapt 3* (crosshatch). Following this, retention was examined by removing visual feedback (gray) for the final 3 blocks (*post 1–3*). *F*: offline ctDCS protocol. ctDCS (anodal/sham) was applied for 25 min during rest between *pre 2* and *adapt 1*. Because of the length of the experiment, retention (no visual feedback blocks) was not examined. G: step adaptation protocol. Following 2 baseline blocks (64 trials: *pre 1* and *pre 2*), a 30° VR was applied to the cursor in steps of 10° per block (96 trials: *adapt 1–3*). A short block (16 trials; explicit) followed this in which participants verbally reported their planned aiming direction. This is thought to measure the participant’s level of cognitive strategy (Taylor et al. 2014). Finally, retention was examined through 1 long block (192 trials) with no visual feedback. *H*: gradual adaptation protocol. A 30° VR was applied to the cursor gradually (0.156° per trial) across 192 trials. It was then maintained at 30° for 96 trials (adapt). A short block (16 trials; explicit) followed this in which participants verbally reported their planned aiming direction. Finally, retention was examined through one long block (192 trials) with no visual feedback.

Participants were seated, with their chin supported by a rest, in front of a computer monitor (30-in., 1,280 × 1,024 pixel resolution, 105 cm from chin rest). A Polhemus motion tracking system (Colchester, VT) was attached to their pronated right index finger, and their arm was placed underneath a horizontally suspended wooden board, which prevented direct vision of the arm (Fig. 1, *A* and *C*). This was unlike the original Galea et al. (2011) study, where participants used a digitized pen and wore goggles to prevent vision of their hand. The visual display consisted of a 1-cm-diameter starting box, a green cursor (0.25-cm diameter) representing the position of their index finger, and a circular white target (0.33-cm diameter). For all experiments, targets appeared in 1 of 8 positions (45° apart) arrayed radially at 8 cm from the central start position. Targets were displayed pseudorandomly so that every set of eight consecutive trials (an “epoch”) included one movement toward each target position. Participants controlled the green cursor on the screen by moving their right index finger across the table (Fig. 1*A*). At the beginning of each trial, participants were asked to move their index finger to the start position, and a target then appeared. Participants were instructed to make a fast “shooting” movement through the target such that online corrections were effectively prevented. At the moment the cursor passed through the invisible boundary circle (an invisible circle centered on the starting position with an 8-cm radius), the cursor was hidden and the intersection point was marked with a yellow square to denote the terminal (end point) error. In addition, a small square icon at the top of the screen changed color on the basis of movement speed. If the movement was completed within 100–300 ms, then it remained white. If the movement was slower than 300 ms, then the box turned red (too slow). Importantly, the participants were reminded that spatial accuracy was the main goal of the task. After each trial subjects moved back to the start, with the cursor only reappearing once they were within 2 cm of the central start position.

#### Cerebellar transcranial direct current stimulation.

Anodal tDCS was delivered (neuroConn, Ilumenau, Germany) through two 5 × 5-cm^2^ electrodes soaked in a saline solution (Wagner et al. 2014). The anodal electrode was placed over the right cerebellar cortex, 3 cm lateral to the inion. The cathodal electrode (reference) was placed over the right buccinator muscle (Galea et al. 2011). At the onset of stimulation, current was increased in a ramplike fashion over a period of 10 s. In the anodal groups, a 2-mA current (current density 0.08 A/cm^2^) was applied for up to 25 min. Because adaptation involved additional trials, cerebellar tDCS was applied for ~8 min longer than in the original study (Galea et al. 2011). In the sham groups, tDCS was ramped up over a period of 10 s and remained on for a further 10 s before being ramped down over 10 s. Participants were blinded to whether they received anodal or sham tDCS (Table 1).

#### Experiment 1: vertical screen.

The aim of *experiment 1* was to replicate the findings of Galea et al. (2011). However, unlike the original Galea et al. (2011) study, participants did not use a digitizing pen and did not wear goggles to prevent vision of their hand. Twenty-eight participants (8 men; 21 ± 4 yr) were split into two groups (anodal and sham, 14 in each group) and exposed to 8 blocks of 96 trials (1 block = 12 repetitions of the 8 targets) during a reaching task in which the computer screen was placed in a vertical position (Fig. 1*A*). The first two blocks acted as baseline and consisted of veridical feedback with (*pre 1*) and without (*pre 2*) online visual feedback. During the trials with no visual feedback, the target was visible, but once the subjects had moved out of the starting position, the cursor indicating their hand position was hidden. In addition, subjects did not receive terminal feedback. Participants were instructed to continue to strike through the target. After this, participants were exposed to three blocks (*adapt 1–3*) of trials in which an abrupt 30° counterclockwise (CCW) visual rotation (VR) was applied. Finally, to assess retention, three blocks (*post 1–3*) were performed without visual feedback. TDCS was applied from the start of *pre 2* until the end of *adapt 3* and lasted for ~25 min (Fig. 1*E*).

#### Experiment 2: horizontal screen.

A large proportion of motor learning studies have been performed while the visual feedback is provided in the same plane as the movement (e.g., Shabbott and Sainburg 2010). Therefore, in *experiment 2* we investigated whether the positive influence of ctDCS on visuomotor adaptation was observed when the screen orientation was flipped to a horizontal position (Fig. 1*B*). Twenty participants (5 men; 22 ± 4 yr) were split into two groups (anodal and sham, 10 in each group) and experienced an experimental protocol identical to that in *experiment 1* (Fig. 1*E*), except that now the participants pointed with their semipronated right index finger underneath a horizontally suspended mirror. The mirror prevented direct vision of the hand and arm but showed a reflection of a computer monitor mounted above that appeared to be in the same plane as the finger (Fig. 1*B*). Once again, participants controlled a cursor on the screen by moving their finger across the table.

#### Experiment 3: tool use.

Several visuomotor studies have required participants to hold a digitizing pen instead of a sensor attached to their finger (Galea et al. 2011; Schlerf et al. 2012). Therefore, in *experiment 3* we changed the motion tracking arrangement so that the Polhemus sensor was attached to the bottom of a pen-shaped tool (Fig. 1*D*). As a result, this was a closer replication of the task design used in the Galea et al. (2011) study than *experiment 1*. However, unlike in the Galea et al. (2011) study, participants did not wear googles that restricted vision of the hand. Twenty-seven subjects (2 men; 21 ± 4 yr) were split into two groups (14 anodal and 13 sham) and experienced an experimental protocol identical to that in *experiment 1* (Fig. 1*E*; vertical screen), except that now participants controlled the cursor on the screen by holding the “pen” and moving it across the surface of the table (Fig. 1*D*).

#### Experiment 4: offline cerebellar tDCS.

Previous work has applied anodal ctDCS during rest and found both physiological and behavioral changes after the cessation of stimulation (Galea et al. 2009; Pope and Miall 2012). This indicates that anodal ctDCS applied during rest (offline ctDCS) could have a beneficial effect on visuomotor adaptation tested after the cessation of stimulation. To examine this, 24 participants (7 men; 20 ± 4 yr) were split into 2 groups (anodal and sham, 12 in each group) and experienced a 25-min rest period between *pre 2* and *adapt 1*. During this time, offline anodal ctDCS was applied (Fig. 1*F*) while participants sat quietly and kept their eyes open. To maintain a similar overall task length, retention (no visual feedback) was not assessed. All other task parameters (vertical screen, tDCS montage) were identical to those in *experiment 1*.

#### Experiments 5 and 6: step and gradual perturbation schedules.

Visuomotor adaptation involves multiple learning mechanisms whose contribution to performance is determined by the task parameters (McDougle et al. 2015). For instance, McDougle et al. suggest that large abrupt visual rotations reduce cerebellum-dependent learning from sensory prediction errors and enhance strategic learning (development of a cognitive plan). In contrast, smaller gradual visual rotations are thought to bias responses toward sensory prediction error learning. If true, then ctDCS should have a particularly beneficial effect on adaptation when the 30° visual rotation is introduced through either multiple small steps (visual rotation introduced in 3 steps of 10°; *experiment 5*) or a gradual paradigm (visual rotation introduced gradually by 0.156° per trial; *experiment 6*).

For *experiment 5*, 36 participants (1 man; 20 ± 1 yr) were split into 2 groups (anodal and sham, 18 in each group). Following 2 baseline blocks (64 trials) with (*pre 1*) and without (*pre 2*) visual feedback, 3 adaptation blocks (96 trials; *adapt 1–3*) exposed participants to a 10°, 20°, and 30° CCW visual rotation (Fig. 1*G*). To examine the degree of cognitive strategy used by each participant, we included a task developed by Taylor et al. (2014). Specifically, following *adapt 3*, participants were asked to verbally report the direction they were aiming toward (Fig. 1*G*, explicit). For these trials (16 in total), the target was presented at the center of a semicircular arc of numbers displayed at 5° intervals. Those CW of the central target were labeled with negative numbers from 1 to 19, and those CCW of the central target were positive numbers from 1 to 19. Participants were asked to report which number they were planning to move their finger toward (Bond and Taylor 2015; McDougle et al. 2015; Taylor et al. 2014). Once participants had provided this verbal response, the numbers disappeared and the participants performed the reaching movement without visual feedback. If a participant was fully aware of the visual rotation, they would report reaching toward number −6 (30° CW), whereas if they were unaware, participants would report aiming to 0 despite moving their finger 30° CW. Finally, a single block (192 trials) without visual feedback examined retention (post).

For *experiment 6*, 32 participants (4 men; 19 ± 1 yr) were split into 2 groups (anodal and sham, 16 in each group). Following 2 baseline blocks (64 trials) with (*pre 1*) and without (*pre 2*) visual feedback, 1 long adaptation block (288 trials; *adapt 1*) involved the 30° CCW visual rotation being applied at rate of 0.156° per trial over 192 trials (Fig. 1*H*). The rotation was then maintained at 30° for a further 96 trials. Participants’ level of cognitive strategy was again assessed (16 trials; explicit) after adaptation. Following this, 1 block of 192 trials without visual feedback examined retention (post).

#### Experiment 7: experiment 1 validation.

Finally, we aimed to validate the results of *experiment 1* by using the same task parameters in a new set of participants. Therefore, 26 participants (7 men; 21 ± 4 yr) were split into two groups (anodal and sham, 13 in each group) and exposed to the same protocol utilized in *experiment 1*.

#### Data analysis.

The 2-D index finger (*X* and *Y*) position data were collected at 120 Hz. For each trial, angular hand direction (°) was calculated as the difference between the angular hand position and angular target position at the point when the cursor intersected an 8-cm invisible circle centered on the starting position. During veridical feedback, the goal was for hand direction to be 0°. However, with a visuomotor rotation, hand direction had to compensate; that is, for a −30° (CCW) visuomotor rotation, a hand direction of +30° (CW) relative to the target was required. Positive values indicate a CW direction, whereas negative values indicate a CCW direction. In addition, reaction time (RT; difference between target appearing and participant moving out of start position) and movement time (MT; difference between reaction time and movement end) were calculated for each trial. We removed any trial in which hand direction, RT, or MT exceeded 2.5 SD above the group mean. This accounted for 8.78 ± 3.04% of trials. One participant in *experiment 4* was removed from the study as a result of failing to follow the task instructions.

Epoch averages were created by binning eight consecutive movements, one toward each target. For each participant, average hand direction was calculated for each target position for *pre 1* (vision baseline) and *pre 2* (no vision baseline). These values were then subtracted to trial-by-trial performance to that particular target in each visual feedback condition (Δhand direction). Specifically, *pre 1* was subtracted away from adaptation performance and *pre 2* was subtracted away from retention performance. For baseline, we averaged hand direction across all epochs of *pre 1* and *pre 2* and compared the anodal and sham groups using two-tailed independent sample *t*-tests. For adaptation, we initially compared Δhand direction in the first trial of *adapt 1* to ensure all participants experienced a similar initial error in response to the visuomotor rotation. We then calculated an average across all the epochs of adaptation excluding *epoch 1*. We believe this best represented the total amount of adaptation expressed by each participant. For retention, we averaged Δhand direction across all the epochs of retention. For each experiment, the anodal and sham groups were compared using two-tailed independent sample *t*-tests. The threshold for all statistical comparisons was *P* < 0.05. Effect sizes are reported as Cohen’s *d*. All data presented are means ± SE, unless otherwise specified. Data and statistical analyses were performed using MATLAB (The MathWorks, Natick, MA) and SPSS (IBM, Armonk, NY).

## RESULTS

### 

#### Experiment 1: vertical screen.

**Fig. 2.**
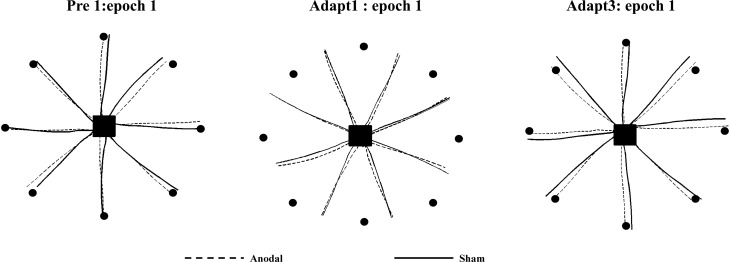
Kinematics data for 2 sample participants in *experiment 1*. Both participants performed similarly during *pre 1* (*left*). In addition, they showed similar initial error when exposed to the 30° CCW visual rotation (*middle*). However, by the end of adaptation, the participants in the anodal group displayed a reduced amount of error in their movement trajectories (*right*).

**Fig. 3.**
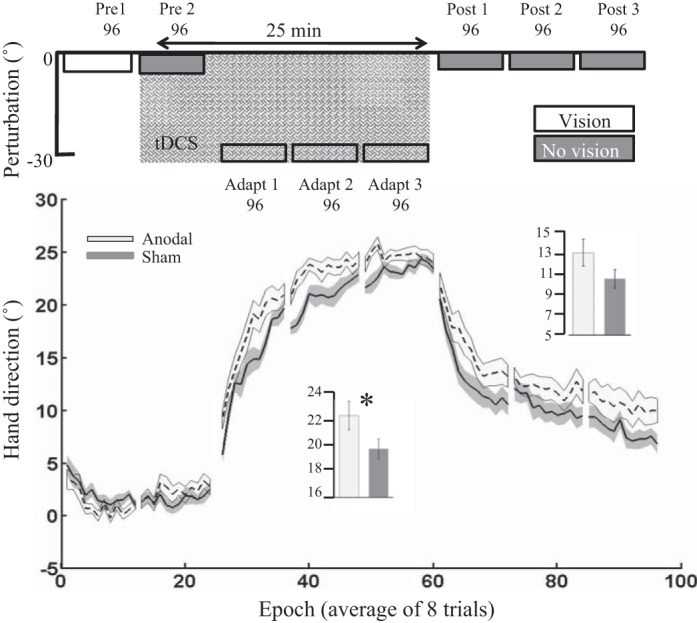
*Experiment 1*: vertical screen. Epoch (average across 8 trials) uncorrected angular hand direction (°) data are shown for the anodal and sham ctDCS groups. Positive values indicate CW hand direction. Bar graph *insets* indicate mean hand direction for the anodal and sham groups during adaptation (*adapt 1–3*) and retention (*post 1–3*). This was determined for each participant by averaging consecutive epochs (see materials and methods). Independent *t*-tests were used to compare values between groups. Solid lines indicate the mean; shaded areas and error bars indicate SE. There was a significant difference between the anodal and sham ctDCS groups (14 in each group) during adaptation [*t*_(26)_ = 2.9, *P = 0.007, *d* = 1.17].

Despite a slightly different setup from that of Galea et al. (2011), we showed that anodal ctDCS led to a greater amount of adaptation relative to sham ctDCS (Figs. 2 and 3). First, both groups behaved similarly during baseline with there being no significant differences between groups during *pre 1* or *pre 2* (Table 2). In addition, when initially exposed to the 30° VR, both groups showed a similar level of performance during the first trial of *adapt 1* (Table 2). However, following this, the anodal group displayed a greater amount of adaptation to the VR compared with the sham group [*t*_(26)_ = 2.9, *P* = 0.007, *d* = 1.17]. Retention in the anodal group appeared to be greater than in the sham group; however, this did not reach statistical significance [*t*_(26)_ = 1.2, *P* = 0.24, *d* = 0.4]. There were no significant differences between groups for either RT or MT during adaptation or retention (Table 3).

**Table 2.** Hand direction in both baselines and change in hand direction (corrected to baseline) in the first trial of adapt 1 are shown across the whole experiments and independent t-test between two groups of anodal and sham

	*Pre 1*	*Pre 2*	1st Trial of *Adapt 1*
*Experiment 1*			
Anodal	0.98 ± 0.97	2.03 ± 2.06	0.3 ± 3.7
Sham	1.91 ± 1.7	1.96 ± 1.8	1.7 ± 7.1
*t*-test	*t*_(26)_ = −1.7, *P* = 0.1	*t*_(26)_ = 1.01, *P* = 0.3	*t*_(26)_ = −0.7, *P* = 0.5
*Experiment 2*			
Anodal	−0.74 ± 0.71	−1.18 ± 1.04	−2.3 ± −2.2
Sham	−0.88 ± 1.06	−1.77 ± 1.40	0.3 ± 3.1
*t*-test	*t*_(18)_ = 0.34, *P* = 0.7	*t*_(18)_ = 1.05, *P* = 0.3	*t*_(18)_ = −1.9, *P* = 0.07
*Experiment 3*			
Anodal	1.07 ± 0.85	2.1 ± 1.52	−0.02 ± 4.2
Sham	1.8 ± 1.8	1.3 ± 1.95	−1.07 ± 4.5
*t*-test	*t*_(25)_ = −1.3, *P* = 0.20	*t*_(25)_ = 1.15, *P* = 0.26	*t*_(25)_ = 0.7, *P* = 0.5
*Experiment 4*			
Anodal	2.4 ± 1.02	1.9 ± 1.03	2.6 ± 5.1
Sham	1.4 ± 0.95	0.39 ± 1.2	−0.3 ± 5.3
*t*-test	*t*_(21)_ = 2. 4, *P* = 0.03*	*t*_(21)_ = 3.2, *P* = 0.003†	*t*_(21)_ = 1.05, *P* = 0.3
*Experiment 5*			
Anodal	0.96 ± 0.91	1.5 ± 1.6	7.4 ± 5.4
Sham	1.2 ± 1.1	2.1 ± 1.9	5.7 ± 5.5
*t*-test	*t*_(34)_ = −0.73, *P* = 0.47	*t*_(34)_ = −0.86, *P* = 0.39	*t*_(34)_ = 0.9, *P* = 0.4
*Experiment 6*			
Anodal	2.04 ± 1.4	1.7 ± 1.6	0.6 ± 5.1
Sham	0.89 ± 1.4	1.5 ± 2.3	3.9 ± 5.0
*t*-test	*t*_(30)_ = 2.3, *P* = 0.03*	*t*_(30)_ = −0.40, *P* = 0.87	*t*_(30)_ = −1.8, *P* = 0.07
*Experiment 7*			
Anodal	1.01 ± 0.9	2.1 ± 1.8	5.1 ± 3.8
Sham	1.4 ± 1.2	2.36 ± 2.1	3.3 ± 3.6
*t*-test	*t*_(24)_ = −0.87, *P* = 0.39	*t*_(24)_ = −0.25, *P* = 0.80	*t*_(24)_ = 0.1, *P* = 0.9

Data are hand direction in both baselines and Δhand direction (corrected to baseline) in the first trial of *adapt 1* shown across whole experiments with independent *t*-tests used to compare anodal and sham groups. Values are means ± SD.

* *P* < 0.05;

† P < 0.01.

**Table 3.** Reaction time and movement time across all experiments

	Reaction Time, s			Movement Time, s		
	Anodal	Sham	*t*-test	Anodal	Sham	*t*-test
*Experiment 1*						
Adaptation	0.38 ± 0.04	0.39 ± 0.06	*t*_(26)_ = 0.24, *P* = 0.8	0.38 ± 0.04	0.38 ± 0.05	*t*_(26)_ = 0.24, *P* = 0.8
Retention	0.37 ± 0.05	0.37 ± 0.05	*t*_(26)_ = 0.08, *P* = 0.9	0.23 ± 0.04	0.22 ± 0.05	*t*_(26)_ = −0.05, *P* = 0.9
*Experiment 2*						
Adaptation	0.49 ± 0.12	0.45 ± 0.02	*t*_(18)_ = 0.8, *P* = 0.4	0.25 ± 0.02	0.25 ± 0.01	*t*_(18)_ = 0.1, *P* = 0.9
Retention	0.44 ± 0.1	0.42 ± 0.02	*t*_(18)_ = 0.5, *P* = 0.6	0.23 ± 0.01	0.23 ± 0.01	*t*_(18)_ = 0.8, *P* = 0.8
*Experiment 3*						
Adaptation	0.39 ± 0.03	0.39 ± 0.04	*t*_(25)_ = −0.19, *P* = 0.8	0.22 ± 0.02	0.22 ± 0.07	*t*_(25)_ = −0.36, *P* = 0.7
Retention	0.39 ± 0.04	0.38 ± 0.04	*t*_(25)_ = 0.43, *P* = 0.7	0.19 ± 0.02	0.21 ± 0.06	*t*_(25)_ = −1.34, *P* = 0.2
*Experiment 4*						
Adaptation	0.45 ± 0.07	0.47 ± 0.02	*t*_(21)_ = −0.5, *P* = 0.6	0.20 ± 0.01	0.20 ± 0.04	*t*_(21)_ = −0.2, *P* = 0.8
*Experiment 5*						
Adaptation	0.40 ± 0.02	0.41 ± 0.02	*t*_(34)_ = −0.3, *P* = 0.7	0.26 ± 0.01	0.27 ± 0.01	*t*_(34)_ = −0.4, *P* = 0.7
Retention	0.39 ± 0.02	0.40 ± 0.01	*t*_(34)_ = −0.6, *P* = 0.5	0.23 ± 0.01	0.23 ± 0.02	*t*_(34)_ = −0.1, *P* = 0.9
*Experiment 6*						
Adaptation	0.35 ± 0.02	0.38 ± 0.02	*t*_(30)_ = −0.7, *P* = 0.5	0.28 ± 0.02	0.30 ± 0.02	*t*_(30)_ = −0.6, *P* = 0.6
Retention	0.34 ± 0.03	0.39 ± 0.02	*t*_(30)_ = −1.4, *P* = 0.2	0.28 ± 0.04	0.22 ± 0.01	*t*_(30)_ = 1.5, *P* = 0.1
*Experiment 7*						
Adaptation	0.44 ± 0.08	0.40 ± 0.05	*t*_(36)_ = 0.9, *P* = 0.1	0.22 ± 0.04	0.23 ± 0.03	*t*_(36)_ = −0.36, *P* = 0.7
Retention	0.42 ± 0.07	0.39 ± 0.04	*t*_(36)_ = 0.4, *P* = 0.2	0.20 ± 0.04	0.21 ± 0.04	*t*_(36)_ = −1.34, *P* = 0.2

Values are means ± SD.

#### Experiment 2: horizontal screen.

**Fig. 4.**
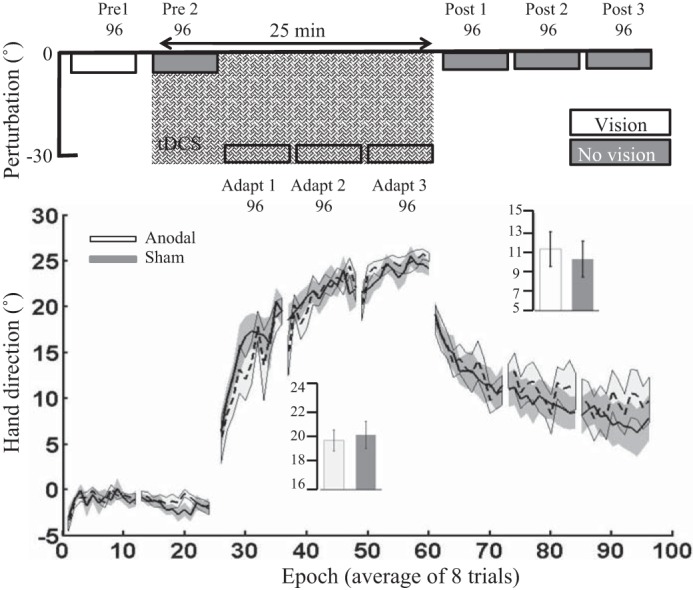
*Experiment 2*: horizontal screen. Epoch (average across 8 trials) uncorrected angular hand direction (°) data are shown for the anodal and sham groups. Positive values indicate CW hand direction. Bar graph *insets* indicate mean hand direction for the anodal and sham groups during adaptation (*adapt 1–3*) and retention (*post 1–3*). This was determined for each participant by averaging consecutive epochs (see materials and methods). Independent *t*-tests were used to compare values between groups. Performance of both groups was identical. Solid lines indicate the mean; shaded areas and error bars indicate SE. There was no significant difference between the anodal and sham ctDCS groups (10 in each group) during adaptation [*t*_(18)_ = −0.005, *P* = 0.9, *d* = 0.00].

In *experiment 2*, an identical stimulation and testing protocol to *experiment 1* was used; however, now the visual feedback was in the same plane as the movement (horizontal screen). Surprisingly, anodal ctDCS was no longer associated with greater adaptation (Fig. 4). First, we found no significant differences between groups for *pre 1*, *pre 2*, or the first trial of *adapt 1* (Table 2). In addition, there were no significant differences between the anodal or sham groups during adaptation [*t*_(18)_ = −0.005, *P* = 0.9, *d* = 0.00; Fig. 4] or retention [*t*_(18)_ = 0.39, *P* = 0.69, *d* = 0.14]. Finally, there were no significant differences between groups for either RT or MT during adaptation or retention (Table 3).

#### Experiment 3: tool use.

**Fig. 5.**
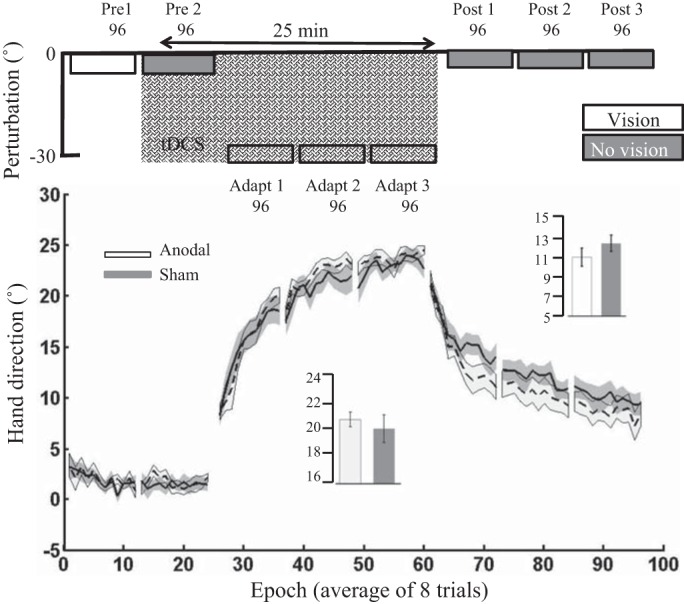
*Experiment 3*: tool use. Epoch (average across 8 trials) uncorrected angular hand direction (°) data are shown for the anodal and sham groups. Positive values indicate CW hand direction. Bar graph *insets* indicate mean hand direction for the anodal and sham groups during adaptation (*adapt 1–3*) and retention (*post 1–3*). This was determined for each participant by averaging consecutive epochs (see materials and methods). Independent *t*-tests were used to compare values between groups. Solid lines indicate the mean; shaded areas and error bars indicate SE. There was no significant difference between the anodal and sham ctDCS groups (14 anodal, 13 sham) during adaptation [*t*_(25)_ = −0.28, *P* = 0.78, *d* = 0.09].

In *experiment 3*, participants once again experienced a protocol identical to that in *experiment 1*; however, instead of performing the task with the sensor attached to their index finger, they held a digitizing pen. This experimental manipulation led to the anodal and sham ctDCS groups behaving similarly across all experimental blocks (Fig. 5). Specifically, there were no significant differences between groups during *pre 1*, *pre 2*,or the first trial of *adapt 1* (Table 2). In addition, no significant differences were observed during adaptation [*t*_(25)_ = −0.28, *P* = 0.78, *d* = 0.09; Fig. 5] or retention [*t*_(25)_ = −1.15, *P* = 0.13, *d* = 0.6]. Finally, there were also no significant differences between groups for either RT or MT during adaptation or retention (Table 3).

#### Experiment 4: offline cerebellar tDCS.

**Fig. 6.**
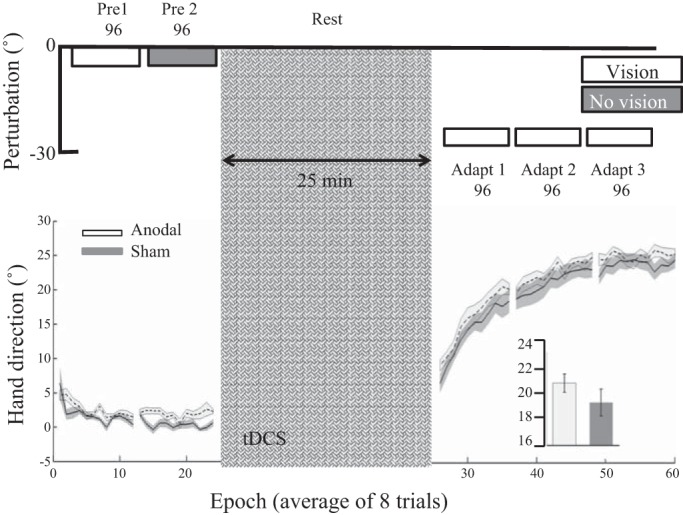
*Experiment 4*: offline cerebellar tDCS. Epoch (average across 8 trials) uncorrected angular hand direction (°) data are shown for the anodal and sham groups. Positive values indicate CW hand direction. Bar graph *insets* indicate mean hand direction for the anodal and sham groups during adaptation (*adapt 1–3*). This was determined for each participant by averaging consecutive epochs. Independent *t*-tests were used to compare values between groups. There was a clear difference between groups during *pre 1*. However, there were no significant differences between groups during adaptation with the use of either hand direction. Solid lines indicate the mean; shaded areas and error bars indicate SE. There was no significant difference between the anodal and sham ctDCS groups (12 anodal, 11 sham) during adaptation [*t*_(21)_ = 0.37, *P* = 0.71, *d* = 0.15].

Next, *experiment 4* examined whether ctDCS applied offline (during 25 min of rest) had a beneficial effect on subsequent visuomotor adaptation. Contrary to our predictions, offline anodal ctDCS did not cause greater adaptation relative to offline sham ctDCS (Fig. 6). Unfortunately, there was a significant difference between groups during *pre 1*, suggesting a small variation (~1°) in baseline performance between groups. However, after baseline correction, there was no significant difference between the anodal and sham ctDCS groups during adaptation [*t*_(21)_ = 0.37, *P* = 0.71, *d* = 0.15]. Finally, there were no significant differences between groups for either RT or MT during adaptation or retention (Table 3). Because of the extended rest period before the adaptation phase (Fig. 6), this experiment did not include a retention block.

#### Experiments 5 and 6: step and gradual perturbation schedules.

**Fig. 7.**
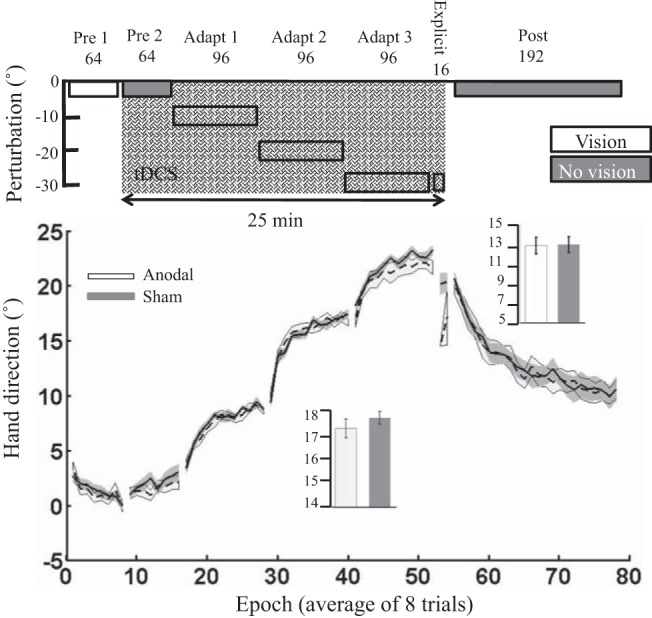
*Experiment 5*: step perturbation schedule. Epoch (average across 8 trials) uncorrected angular hand direction (°) data are shown for the anodal and sham groups. Positive values indicate CW hand direction. Bar graph *insets* indicate mean hand direction for the anodal and sham groups during adaptation (*adapt 1–3*) and retention. This was determined for each participant by averaging consecutive epochs (see materials and methods). Independent *t*-tests were used to compare values between groups. Performance of the anodal and sham groups was identical throughout the experiment. Solid lines indicate the mean; shaded areas and error bars indicate SE. There was no significant difference between the anodal and sham ctDCS groups (18 in each group) during adaptation [*t*_(34)_ = −0.35, *P* = 0.72, *d* = 0.1].

**Fig. 8.**
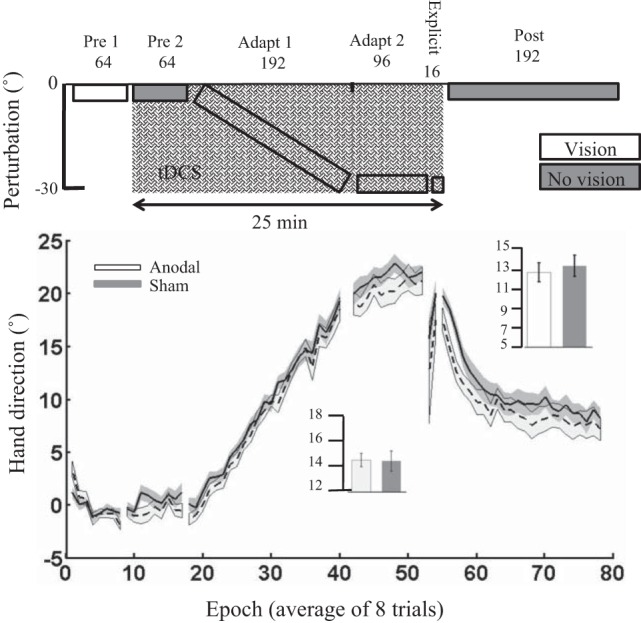
*Experiment 6*: gradual perturbation schedule. Epoch (average across 8 trials) uncorrected angular hand direction (°) data are shown for the anodal and sham groups. Positive values indicate CW hand direction. Bar graph *insets* indicate mean hand direction for the anodal and sham groups during adaptation blocks and retention (post). This was determined for each participant by averaging consecutive epochs (see materials and methods). Independent *t*-tests were used to compare values between groups. Performance of the anodal and sham groups was identical throughout the experiment. Solid lines indicate the mean; shaded areas and error bars indicate SE. There was no significant difference between the anodal and sham ctDCS groups (16 in each group) during adaptation [*t*_(30)_ = 0.1, *P* = 0.9, *d* = 0.00].

Finally, *experiments 5* and *6* tested whether anodal ctDCS was more effective when the 30° visual rotation was introduced with either a stepped (visual rotation introduced in 3 steps of 10°; *experiment 5*) or gradual paradigm (visual rotation introduced gradually by 0.156° per trial; *experiment 6*). However, once again, we found no significant effect of anodal ctDCS on adaptation (Figs. 7 and 8).

In *experiment 5*, there were no significant differences between the anodal and sham groups during *pre 1* or *pre 2*, or when initially exposed to the 10° VR (Table 2). In addition, no significant differences were observed across adaptation [*t*_(34)_ = −0.35, *P* = 0.72, *d* = 0.1; Fig. 7] or retention [*t*_(34)_ = −0.9, *P* = 0.37, *d* = 0.3]. To examine the degree of cognitive strategy used by each participant, after *adapt 3* we asked participants to verbally report the direction they were aiming toward (Fig. 1*G*, explicit). Despite displaying a hand direction of ~20° (Fig. 7), participants in both groups reported a similar aiming direction toward the target [Explicit report anodal: 1.7 ± 2.1°; sham: 1.4 ± 4.1°; independent *t*-test: *t*_(34)_ = 0.47, *P* = 0.64, *d* = 0.09]. This indicates that all participants had developed only a minimal cognitive aiming strategy. During this explicit block, although there was no significant difference between groups for Δhand direction [*t*_(34)_ = −1.8, *P* = 0.07, *d* = 0.61], there did appear to be a trend for the anodal group to display reduced hand direction relative to the sham group (Fig. 7). In addition, there were no significant differences between groups for either RT or MT during adaptation or retention (Table 3).

In *experiment 6*, there was a significant difference between groups during *pre 1* (Table 2), suggesting a small variation (1°) in baseline performance between groups. Again, to account for these differences, we subtracted each participant’s average hand direction during *pre 1* from their subsequent performance, and there was no significant difference between the anodal and sham ctDCS groups during adaptation [*t*_(30)_ = 0.01, *P* = 0.9, *d* = 0.00; Fig. 8] or retention [*t*_(30)_ = −1.00, *P* = 0.3, *d* = 0.35]. Similarly to *experiment 5*, despite displaying a hand direction of ~20° (Fig. 8), participants in both groups reported a similar aiming direction toward the target (Explicit report anodal: 0.64 ± 1.5°; sham: 0.37 ± 0.7°; independent *t*-test: *t*_(30)_ = 0.67, *P* = 0.51, *d* = 0.23]. This indicates that all participants had developed only a minimal cognitive aiming strategy. During this block, there also was no significant difference between groups for actual Δhand direction [*t*_(30)_ = −0.9, *P* = 0.4, *d* = 0.3]. There were no significant differences between groups for either RT or MT during adaptation or retention (Table 3).

#### Experiment 7: experiment 1 validation.

**Fig. 9.**
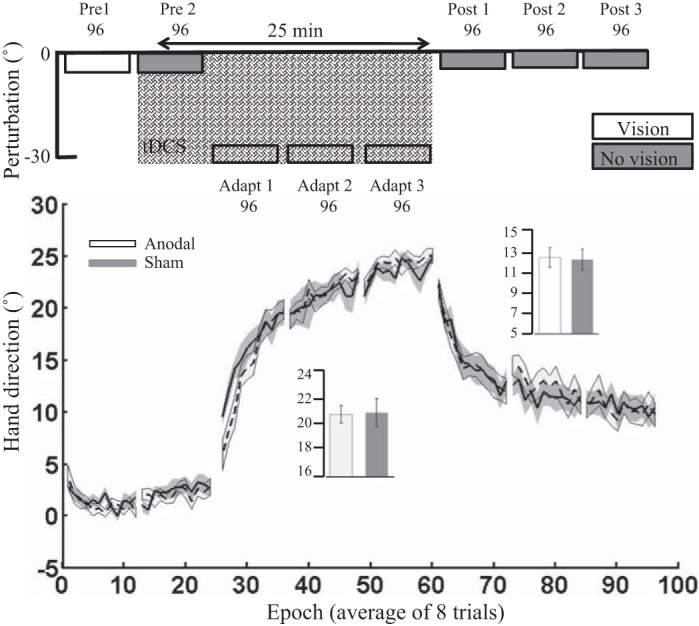
*Experiment 7*: *experiment 1* validation. Epoch (average across 8 trials) uncorrected angular hand direction (°) data are shown for the anodal and sham groups. Positive values indicate CW hand direction. Bar graph *insets* indicate mean hand direction for the anodal and sham groups during adaptation blocks and retention (post). This was determined for each participant by averaging consecutive epochs (see materials and methods). Independent *t*-tests were used to compare values between groups. Performance of the anodal and sham groups was identical throughout the experiment. Solid lines indicate the mean; shaded areas and error bars indicate SE. There was no significant difference between the anodal and sham ctDCS groups (13 in each group) during adaptation [*t*_(24)_ = −2.5, *P* = 0.8, *d* = 0.1].

To validate our only positive result, we repeated *experiment 1* with two new groups (anodal and sham) of naive participants. Unfortunately, we found no significant difference between the anodal and sham ctDCS groups. There were no significant differences between groups during *pre 1* or *pre 2*, or when initially exposed to the 30° VR (Table 2). In addition, there were no differences between groups across adaptation [*t*_(24)_ = −2.5, *P* = 0.8, *d* = 0.1; Fig. 9] or retention [*t*_(24)_ = 0.23, *P* = 0.8, *d* = 0.1]. Finally, there were no significant differences between groups for either RT or MT during adaptation or retention (Table 3).

Despite the differences between the current experimental set up and Galea et al. (2011), such as number of trials, duration of tDCS, and use of tool, we pooled data across *experiments 1* and *2* from Galea et al. (2011) and *experiments 1*, *3*, and *7* from the current study. For each participant, we calculated an average Δhand direction across all adaptation epochs, excluding *epoch 1*, and performed an independent *t*-test between the pooled anodal (*n* = 61) and sham (*n* = 60) groups. These pooled data showed a significant difference between anodal (20.1 ± 2.9) and sham ctDCS [17.5 ± 4.1; *t*_(119)_ = 3.9, *P* = 0.0005, *d* = 0.7]. Interestingly though, the effect size was substantially smaller than the positive results found in *experiment 1*.

#### Self-reported ratings of attention, fatigue, and sleep.

There were no significant differences between groups across all experiments for the self-reported ratings of attention, fatigue, and quality of sleep (Table 1).

## DISCUSSION

Across all seven experiments, participants showed a clear ability to adapt to the novel visuomotor rotation. In *experiment 1*, we were able to show that anodal cerebellar tDCS caused a greater amount of adaptation relative to sham tDCS; however, this did not hold when we repeated the same experiment with a new set of participants (*experiment 7*). Although similar, these experiments differed from the original Galea et al. (2011) study in which participants used a digitized pen and wore goggles to prevent vision of the hand. When manipulating experimental parameters such as screen orientation (*experiment 2*), use of a tool (*experiment 3*), tDCS timing (*experiment 4*), and the perturbation schedule (*experiments 5* and *6*), we found anodal cerebellar tDCS to have no effect on visuomotor adaptation.

### 

#### tDCS did not enhance visuomotor adaptation when a horizontal screen was used.

Although the facilitatory effect of cerebellar tDCS on motor learning has been shown across visuomotor adaptation (Galea et al. 2011), force field adaptation (Herzfeld et al. 2014), locomotor adaptation (Jayaram et al. 2012), saccade adaptation (Avila et al. 2015; Panouillères et al. 2015), motor skill learning (Cantarero et al. 2015), and language prediction tasks (Miall et al. 2016), the sensitivity of this effect to specific task parameters had not been previously documented. Because a large proportion of motor learning studies are performed while the visual feedback is provided in the same plane as the movement (Herzfeld et al. 2014; Shabbott and Sainburg 2010), we were first motivated to examine whether the positive influence of tDCS on visuomotor adaptation can be observed when the screen orientation was flipped to a horizontal position. Thus *experiments 1* and *2* addressed this issue by first replicating the screen display used in Galea et al. (2011) and then showing that tDCS was not associated with greater adaptation in the more typical in-plane feedback condition. The posterior part of the cerebellum is important for visuomotor adaptation (Rabe et al. 2009) and heavily connected with the posterior parietal cortex (O’Reilly et al. 2010), which is crucial for visuomotor control (Culham et al. 2006). Because modeling studies suggest cerebellar tDCS mainly activates the posterior part of the cerebellum (Ferrucci et al. 2012; Parazzini et al. 2014; Rampersad et al. 2014), the increased visuomotor complexity and presumed greater reliance on the posterior cerebellum with a vertical screen orientation may optimize the effects of cerebellar tDCS on visuomotor adaptation.

#### tDCS did not improve visuomotor adaptation even when participants used a tool.

Next, we were unable to replicate the original Galea et al. (2011) study where participants held a tool/digitizing pen (Block and Celnik 2013; Galea et al. 2011). Although *experiment 3* was a closer replication of Galea et al. (2011) than *experiments 1* and *7*, participants still did not wear googles to restrict vision of the hand. Although not significant, Fig. 5 does suggest there was a trend toward the anodal tDCS group adapting by a greater amount.

#### tDCS aftereffect did not affect visuomotor adaptation.

It also has been reported that anodal cerebellar tDCS applied during rest can lead to both physiological and behavioral changes over a period of 10–30 min after the cessation of stimulation (Galea et al. 2009; Pope and Miall 2012). This indicates that the aftereffect of cerebellar tDCS could have a beneficial effect on visuomotor adaptation. However, following 25 min of offline anodal cerebellar tDCS, we found no observable differences between the anodal and sham groups. One significant issue is that despite having neurophysiological evidence regarding the changes associated with offline cerebellar tDCS (Galea et al. 2009), no such data exist for its online effects. Therefore, we currently do not know whether the online and offline effects of cerebellar tDCS are consistent or whether one is more potent than the other.

#### tDCS did not enhance adaptation when the perturbation was applied gradually.

The contribution of the cerebellum to abrupt and gradual perturbation paradigms is an area of continued interest within the motor adaptation literature. For example, Criscimagna-Hemminger et al. (2010) showed cerebellar lesion patients were unable to adapt to abrupt perturbations but preserved the capacity to adapt to gradual perturbations. Similarly, Schlerf et al. (2012) reported modulation of cerebellar excitability for abrupt, but not gradual, visuomotor adaptation (Schlerf et al. 2012). However, Gibo et al. 2013 showed that cerebellar lesion patients may use noncerebellar strategic learning to successfully adapt. In line with this argument, other recent work suggested that large abrupt visual rotations reduce cerebellum-dependent sensory prediction error learning and enhance strategic learning, whereas smaller visual rotations bias learning toward sensory prediction error learning (Bond and Taylor 2015; McDougle et al. 2015; Taylor et al. 2014). This suggests that cerebellar tDCS may have been more effective with small or gradual perturbation schedules. However, we found that tDCS did not show any significant effect on adaptation when the perturbation was applied in small steps (*experiment 5*) or gradually (*experiment 6*).

#### The positive effect of cerebellar tDCS in experiment 1 was not replicated.

Finally, we wanted to see whether the positive effect of cerebellar tDCS on visuomotor adaptation observed in *experiment 1* could be replicated in a new set of naive participants. Unfortunately, this positive effect was not observed, with *experiment 7* showing no significant difference between the anodal and sham tDCS groups during adaptation. This suggests that either the positive effects of cerebellar tDCS in *experiment 1* were observed by chance or the effect size of cerebellar tDCS is significantly smaller than one might imagine. Although our sample sizes (10–15 per group) were in the range of those in previously published tDCS papers (Block and Celnik 2013; Cantarero et al. 2015; Galea et al. 2011; Hardwick and Celnik 2014), a recent study indicated this could be significantly under powered (Minarik et al. 2016). Minarik et al. (2016) showed that with a suggested tDCS effect size of 0.45, the likelihood of observing a significant result with 14 participants (per group) was ~20%. To examine this further, we pooled data across *experiments 1* and *2* from Galea et al. (2011) and *experiments 1*, *3*, and *7* from the current study. These pooled data showed a significant difference between anodal and sham ctDCS; however, the effect size was substantially smaller (0.7) than what was initially observed in *experiment 1*. At present it is difficult to determine a true effect size for not only cerebellar tDCS but also tDCS in general due to the clear publication bias toward positive effects in the literature. Through informal discussion with many colleagues, we find it is clear that researchers are observing null effects with cerebellar tDCS but have so far been slow to publish these results. Although this is beginning to change (Mamlins 2016; Steiner et al. 2016; Westwood et al. 2017), we believe a more accurate representation of the effect size, and so the required participant numbers, of cerebellar tDCS will only be achieved if null results are published more often.

Another possible limitation with the current design is the use of a between-subject paradigm. Previous work has shown large interindividual variation in motor learning rates (Stark-Inbar et al. 2017), implementation of motor learning processes (Christou et al. 2016), and responsivity to stimulation (Wiethoff et al. 2014). These factors may all negatively affect our ability to observe consistent between-subject tDCS differences in motor learning. Although a within-subject design would overcome many of these issues, it would also introduce the substantial problem of carry-over effects being observed with visuomotor adaptation weeks after initial exposure (Krakauer 2009).

#### Future direction.

Our results indicate that for cerebellar tDCS to become an effective tool, technical advances must be identified that improve the strength and consistency of its effect on functional tasks. For example, the common assumption is to that currents of 1–2 mA are effective (Woods et al. 2016). However, previous work has used currents of up to 5 mA on other brain areas (Bonaiuto and Bestmann 2015; Furubayashi et al. 2008; Hämmerer et al. 2016), suggesting greater current intensities are possible with cerebellar tDCS. Alternatively, there is exciting work suggesting high-definition tDCS combined with computational modeling of the brain’s impedances can lead to exact predictions regarding the behavioral results associated with tDCS (Bonaiuto and Bestmann 2015; Furubayashi et al. 2008; Hämmerer et al. 2016). It is possible that using high-definition tDCS along with computational modeling to optimize electrode placement could enhance the magnitude and reliability of the tDCS effect on the cerebellum (Kuo et al. 2013).

#### Conclusions.

In conclusion, we failed to find a consistent effect of cerebellar tDCS on visuomotor adaptation. Although we initially replicated previous reports of cerebellar tDCS enhancing visuomotor adaptation, we found this not to be consistent across varying task parameters, nor reproducible in a new group of participants. We believe these results highlight the need for substantially larger group sizes for tDCS studies and may call into question the validity of using cerebellar tDCS within a clinical context where a robust effect across behaviors would be required.

## GRANTS

R. Jalali was supported by a Funds for Women Graduates main grant, R. C. Miall by the Wellcome Trust, and J. M. Galea by European Research Council MotMotLearn Grant 63748.The Physical Sciences of Imaging in the Biomedical Sciences doctoral program is supported by *Engineering and Physical Sciences Research Council* Grant EP/F50053X/1.

## DISCLOSURES

No conflicts of interest, financial or otherwise, are declared by the authors.

## AUTHOR CONTRIBUTIONS

R.J., R.C.M., and J.M.G. conceived and designed research; R.J. performed experiments; R.J. and J.M.G. analyzed data; R.J., R.C.M., and J.M.G. interpreted results of experiments; R.J. prepared figures; R.J. drafted manuscript; R.J., R.C.M., and J.M.G. edited and revised manuscript; R.J., R.C.M., and J.M.G. approved final version of manuscript.
